# Differential Cytotoxicity Mechanisms of Copper Complexed with Disulfiram in Oral Cancer Cells

**DOI:** 10.3390/ijms22073711

**Published:** 2021-04-02

**Authors:** Ssu-Yu Chen, Yung-Lung Chang, Shu-Ting Liu, Gunng-Shinng Chen, Shiao-Pieng Lee, Shih-Ming Huang

**Affiliations:** 1 Department of Biochemistry, National Defense Medical Center, Taipei City 114, Taiwan; windblowvoice@gmail.com (S.-Y.C.); ylc7305@gmail.com (Y.-L.C.); shuting0719@gmail.com (S.-T.L.); 2 School of Dentistry, Department of Dentistry of Tri-Service General Hospital, National Defense Medical Center, Taipei City 114, Taiwan; 3 Department of Biomedical Engineering, National Defense Medical Center, Taipei City 114, Taiwan

**Keywords:** disulfiram, copper, c-Myc, reactive oxygen species, HIF-1α

## Abstract

Disulfiram (DSF), an irreversible aldehyde dehydrogenase inhibitor, is being used in anticancer therapy, as its effects in humans are known and less adverse than conventional chemotherapy. We explored the potential mechanism behind the cytotoxicity of DSF-Cu^+^/Cu^2+^ complexes in oral epidermoid carcinoma meng-1 (OECM-1) and human gingival epithelial Smulow-Glickman (SG) cells. Exposure to CuCl_2_ or CuCl slightly but concentration-dependently decreased cell viability, while DSF-Cu^+^/Cu^2+^ induced cell death in OECM-1 cells, but not SG cells. DSF-Cu^+^/Cu^2+^ also increased the subG1 population and decreased the G1, S, and G2/M populations in OECM-1 cells, but not SG cells, and suppressed cell proliferation in both OECM-1 and SG cells. ALDH enzyme activity was inhibited by CuCl and DSF-Cu^+^/Cu^2+^ in SG cells, but not OECM-1 cells. ROS levels and cellular senescence were increased in DSF-Cu^+^/Cu^2+^-treated OECM-1 cells, whereas they were suppressed in SG cells. DSF-Cu^+^/Cu^2+^ induced mitochondrial fission in OECM-1 cells and reduced mitochondrial membrane potential. CuCl_2_ increased but DSF- Cu^2+^ impaired oxygen consumption rates and extracellular acidification rates in OECM-1 cells. CuCl_2_ stabilized HIF-1α expression under normoxia in OECM-1 cells, and complex with DSF enhanced that effect. Levels of c-Myc protein and its phosphorylation at Tyr58 and Ser62 were increased, while levels of the N-terminal truncated form (Myc-nick) were decreased in DSF-Cu^+^/Cu^2^-treated OECM-1 cells. These effects were all suppressed by pretreatment with the ROS scavenger NAC. Overexpression of c-Myc failed to induce HIF-1α expression. These findings provide novel insight into the potential application of DSF-CuCl_2_ complex as a repurposed agent for OSCC cancer therapy.

## 1. Introduction

Oral squamous cell carcinoma (OSCC) can arise anywhere within the oral cavity, including the buccal mucosa, oral floor, palate, tongue, or upper or lower gingiva [1,2,3]. The molecular pathogenesis of OSCC entails the complex interplay between genetic mutations and altered levels of transcripts, proteins, and metabolites. Moreover, the oral cavity is richly supplied with lymphatic vessels that form numerous anastomoses, which facilitates cervical lymph node metastasis of late-stage OSCC tumors. Consequently, the recurrence potential of OSCC is closely associated with nodal metastasis of the tumor.

Alcohol consumption promotes a disease state microbiome associated with OSCC [4,5]. The effect of this inflammatory composition is heightened by alcohol-induced modulation of the immune response and cell structures. In response to alcohol consumption, microbes that can contribute to genotoxic levels of acetaldehyde through expression of alcohol dehydrogenase also increase reactive oxygen species (ROS) production and promote inflammation. The resulting oral environment, characterized by oxidative stress and inflammation, is susceptible to damage from acetaldehyde and promotes tumorigenesis.

ROS include hydrogen peroxide, superoxide anion and hydroxyl radical [6,7]. The major endogenous sources of ROS are the mitochondrial respiratory chain, NADPH oxidase, and peroxisomes [8]. A moderate increase in ROS balanced with antioxidant capacity favors cell proliferation and survival. However, when ROS levels are high enough to overwhelm the cell’s antioxidant capacity, cell death is triggered by various oxidizing macromolecules and metabolites [9]. Growing evidence suggests that cancer cells exhibit greater intrinsic oxidative stress or ROS levels than healthy cells due to metabolic abnormalities and oncogenic signaling [7]. Higher ROS levels activate the redox signaling required for survival and promote tumorigenesis, though cancer cells also maintain an inducible and active antioxidant defense system to protect themselves from ROS-induced cell death [10]. Notably, some chemotherapeutic drugs currently in use, including doxorubicin and cisplatin, increase ROS stress to overcome the anti-oxidative capacity of cancer cells, resulting in cell death [9,11].

For about 70 years disulfiram (DSF) has been used as an irreversible aldehyde dehydrogenase (ALDH) inhibitor [12,13]. Its cost effectiveness and fewer adverse effects are advantages that DSF has over conventional cancer chemotherapeutic drugs. Other important characteristics of DSF are its ability to inhibit proteasome function and suppress various cancer-associated mediators, including ROS, PIK, MAPK, NF-κB, ALDH, EGFR/Src/VEGF, and others [14,15,16]. DSF also acts as a metal chelator that primarily complexes with Cu^2+^ [17].

The Fenton reaction, catalyzed by Cu^+^/Cu^2+^ or Fe^2+^/Fe^3+^, produces reactive hydroxyl radicals leading to severe oxidative damage [18]. Increased oxidative stress leads to protein oxidation that releases copper ions from proteins and reduces levels of glutathione-bound Cu^+^. Cu^2+^ is generally reduced by superoxide and hydroascorbate to complete the catalytic cycle [19]. Excess copper ions may displace other metals from their cognate ligands in many important enzymes, such as specific prolyl hydroxylases, for hypoxia-inducible factor-1 alpha (HIF-1α) protein degradation, resulting in impairment of their enzymatic activities [20]. This reaction requires oxygen, 2-oxoglutarate, ascorbate, and Fe^2+^. Similar to the competition between Fe^2+^ (a cofactor for prolyl hydroxylase activity) and Cu ions, ROS alters the oxidation state of Fe^2+^ to Fe^3+^, making it unusable for prolyl hydroxylases, thereby promoting HIF-1α stabilization [21].

HIF-1 functions as a transcription factor that mediates adaptive responses to changes in tissue oxygenation [22]. HIF-1α synthesis is regulated via O_2_-independent mechanisms, but HIF-1α degradation is regulated primarily via O_2_-dependent mechanisms. More than 60 putative HIF-1 target genes involved in angiogenesis, cell survival, glucose metabolism, and invasion, have been identified. c-Myc is a transcription factor involved in the regulation of cellular growth, differentiation, apoptosis, and metabolism, acting via a protein–protein network to activate or repress transcription of its target genes [23,24]. HIF-1α and c-Myc apparently have opposing functions in cell proliferation, mitochondrial biogenesis, and DNA repair, acting via shared target genes, such as those involved in glycolysis and angiogenesis [25,26]. HIF-1α regulates cell cycle and DNA repair genes by counteracting c-Myc activities through c-Myc displacement. HIF-1α controls mitochondrial biogenesis by inhibiting c-Myc-mediated transcription through induction of c-Myc degradation and Mxi1 expression. In c-Myc-dysregulated cells, HIF-1α cooperates with c-Myc to enhance common target gene expression. It is a puzzle of functional interaction between c-Myc and HIF-1α in OSCC.

Repurposing old drugs for new applications in cancer therapy is a very attractive and exciting field. In the present study, we complexed DSF with Cu^+^ or Cu^2+^ to differentiate their working mechanism(s) and dissect the potential cytotoxic mechanisms of the DSF-Cu^+^/Cu^2+^ complexes in OSCC. We hope that our findings provide novel insight into the antitumor mechanisms of DSF-Cu^+^/Cu^2+^ in OSCC.

## 2. Results

### 2.1. Effects of Copper, DSF, and DSF-Cu^+^/Cu^2+^ Complexes on Cell Viability, Cell Cycle Prolife, Proliferation, Apoptosis, and Senescence in OECM-1 and SG Cells

**Figure 1:**
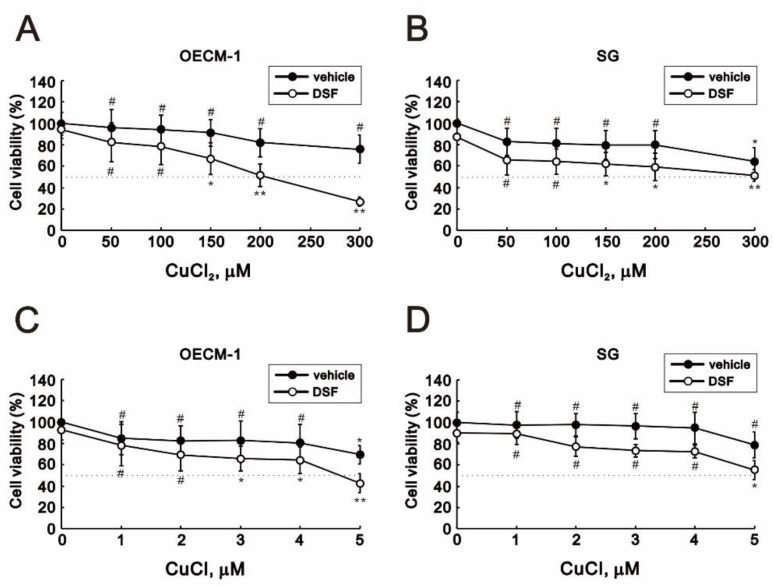
Effects of disulfiram (DSF), copper ions, and DSF-copper complexes on cell viability in OECM-1 and SG cells. (**A**–**D**) OECM-1 (**A**,**C**) and SG (**B**,**D**) cells were treated for 3 h (**A**,**B**) or 6 h (**C**,**D**) with the indicated concentrations of Cu^+^ or Cu^2+^ in the absence or presence of 0.5 μM DSF. Cell viability was measured using MTT assays. The results are representative of three independent experiments. # *p* > 0.05, * *p* < 0.05, and ** *p* < 0.01.

The cytotoxicity of copper and the DSF-Cu^+^/Cu^2+^ complexes has been reported previously [27,28,29]. We therefore applied MTT cell viability assays to assess the effects of Cu^+^, Cu^2+^, and the combination of DSF and Cu^+^ or Cu^2+^ on OECM-1 oral cancer cells and SG normal gingival epithelial cells [30]. We observed that medium supplemented with various concentrations of CuCl and CuCl_2_ induced concentration-dependent cytotoxicity in OECM-1 and SG cells (Figure 1). The combination of DSF with copper ion, especially Cu^2+^, dramatically increased the cytotoxicity toward OECM-1 cells (Figure 1A). The IC_50_’s for CuCl_2_ and CuCl were, respectively, 200 μM and 4.6 μM in OECM-1 cells (Figure 1A,C) and >300 μM and 5 μM in SG cells (Figure 1B,D).

We used 7-AAD cell cycle profile analysis to address the effect of copper ion and DSF-Cu^+^/Cu^2+^ complexes on the cell cycle profile in OECM-1 and SG cells. The combination of DSF with CuCl_2_ or CuCl dramatically increased the subG1 population while decreasing the G1, S, and G2/M populations in OECM-1 cells (Table 1).

**Figure 2:**
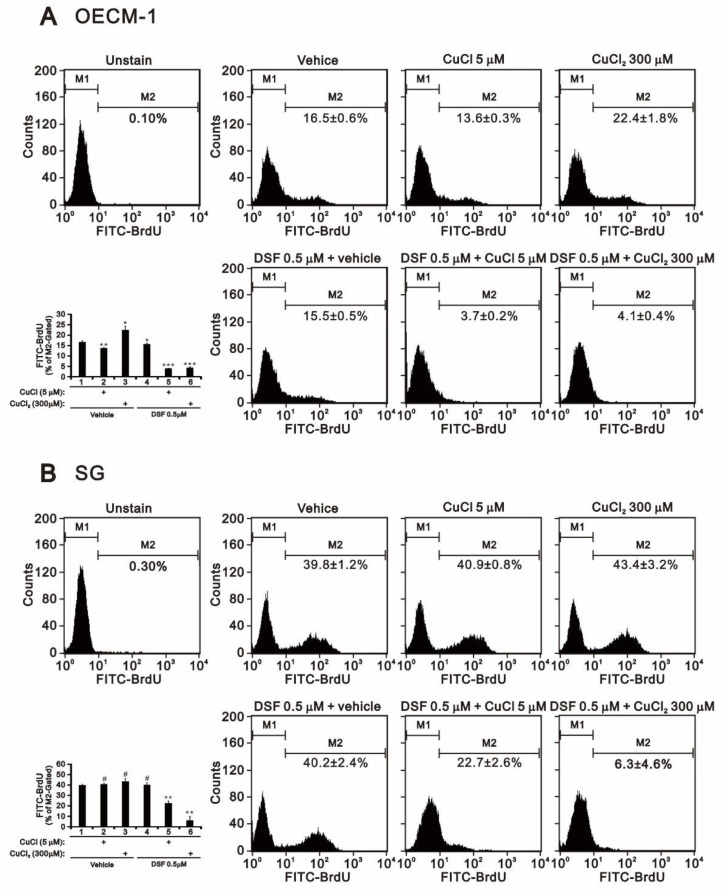
Effects of DSF, copper ions, and DSF-copper complexes on cell proliferation in OECM-1 and SG cells. (**A**) OECM-1 and (**B**) SG cells (2 × 10^5^ cells/well) were treated for 2 h or 4 h with the indicated concentrations of Cu^+^ or Cu^2+^ in the absence or presence of 0.5 μM DSF. They were then subjected to BrdU proliferation analysis. The results are representative of three independent experiments. # *p* > 0.05, * *p* < 0.05, ** *p* < 0.01, and *** *p* < 0.001.

In SG cells, the combination of DSF with CuCl_2_ or CuCl had no effect on the subG1 population but increased the G1 and G2/M populations and dramatically decreased the S population. Given the decrease in the S population, we applied BrdU proliferation analysis to confirm the effect on cell proliferation. Alone, CuCl_2_ or DSF promoted proliferation, whereas CuCl alone suppressed OECM-1 cell proliferation (Figure 2A) and had no effect on SG cells (Figure 2B). The combination of DSF with CuCl_2_ or CuCl decreased both OECM-1 and SG cell proliferation (Figure 2). However, DSF-Cu^2+^ complex had a more suppressive effect on SG cell proliferation than did the DSF-Cu^+^ complex (Figure 2B).

**Figure 3:**
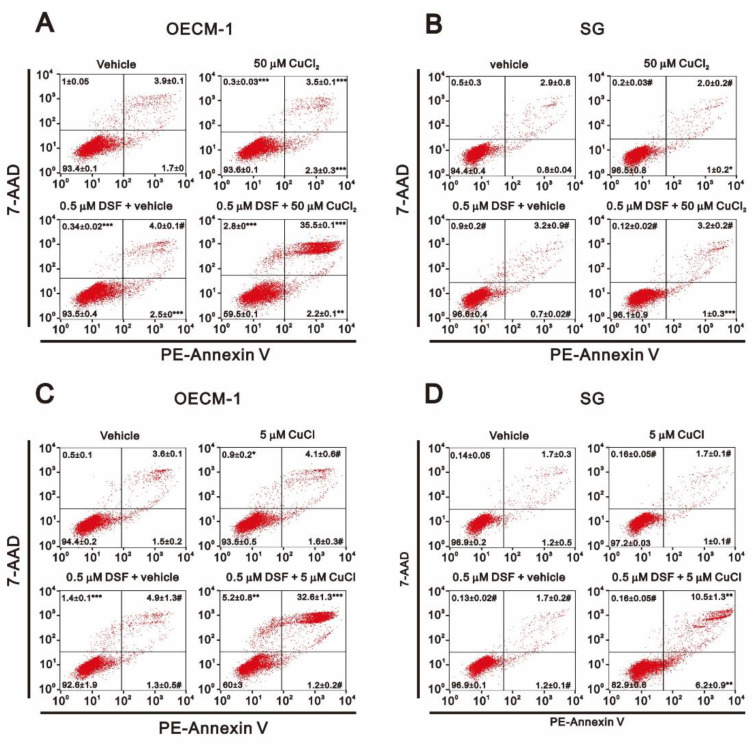
Effects of DSF, copper ions, and DSF-copper complexes on apoptosis in OECM-1 and SG cells. (**A**–**D**) OECM-1 (**A**,**C**) and SG (**B**,**D**) cells were treated for 3 h (**A**,**B**) or 6 h (**C**,**D**) with the indicated concentrations of Cu^+^ or Cu^2+^ in the absence or presence of 0.5 μM DSF. They were then subjected to Annexin V apoptosis analysis. Early apoptotic cells are PE Annexin V positive and 7-AAD negative, while late apoptotic cells are both PE Annexin V and 7-AAD positive. The results are representative of three independent experiments. # *p* > 0.05, * *p* < 0.05, ** *p* < 0.01, and *** *p* < 0.001.

**Figure 4:**
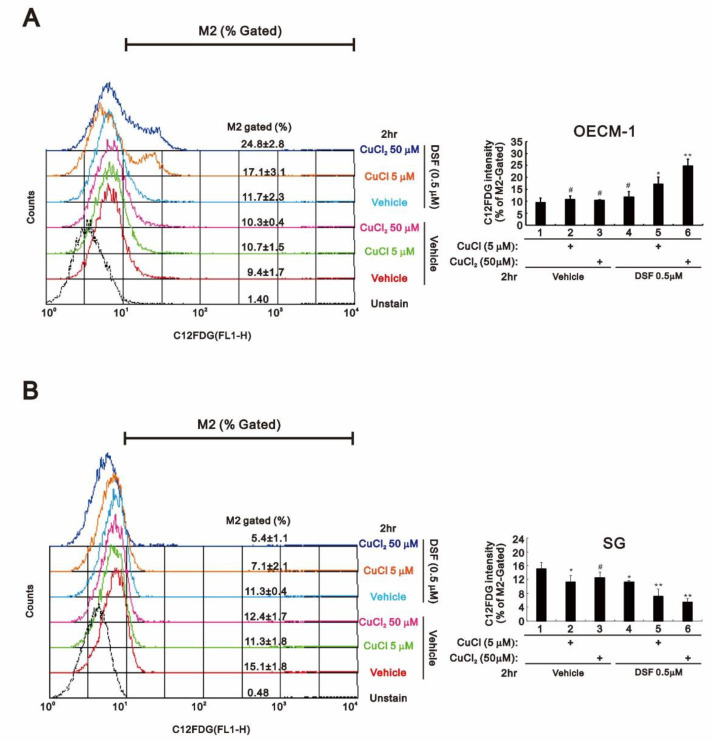
Effects of DSF, copper ions, and DSF-copper complexes on senescence in OECM-1 and SG cells. (**A**) OECM-1 and (**B**) SG cells (2 × 10^5^ cells/well) were treated for 2 h or 4 h with the indicated concentrations of Cu^+^ or Cu^2+^ in the absence or presence of 0.5 μM DSF, after which the live cells were stained with 33 μM C12FDG and assayed using a flow cytometer. The results are representative of three independent experiments. # *p* > 0.05, * *p* < 0.05, and ** *p* < 0.01.

Annexin V apoptosis analysis revealed that the combination of DSF with CuCl_2_ or CuCl dramatically increased the incidence of late apoptosis, but not early apoptosis, among OECM-1 (Figure 3A,C). However, the combination of DSF with CuCl appeared to induce both early and late apoptosis in SG cells (Figure 3D). C12FDG senescence analysis showed that the combination of DSF with CuCl or CuCl_2_ dramatically increased the population of senescent OECM-1 cells (Figure 4A), whereas both combinations decreased the population of senescent SG cells (Figure 4B).

### 2.2. Effects of Copper, DSF and DSF-Cu^+^/Cu^2+^ Complexes on Cytosolic and Mitochondrial ROS in OECM-1 and SG Cells

**Figure 5:**
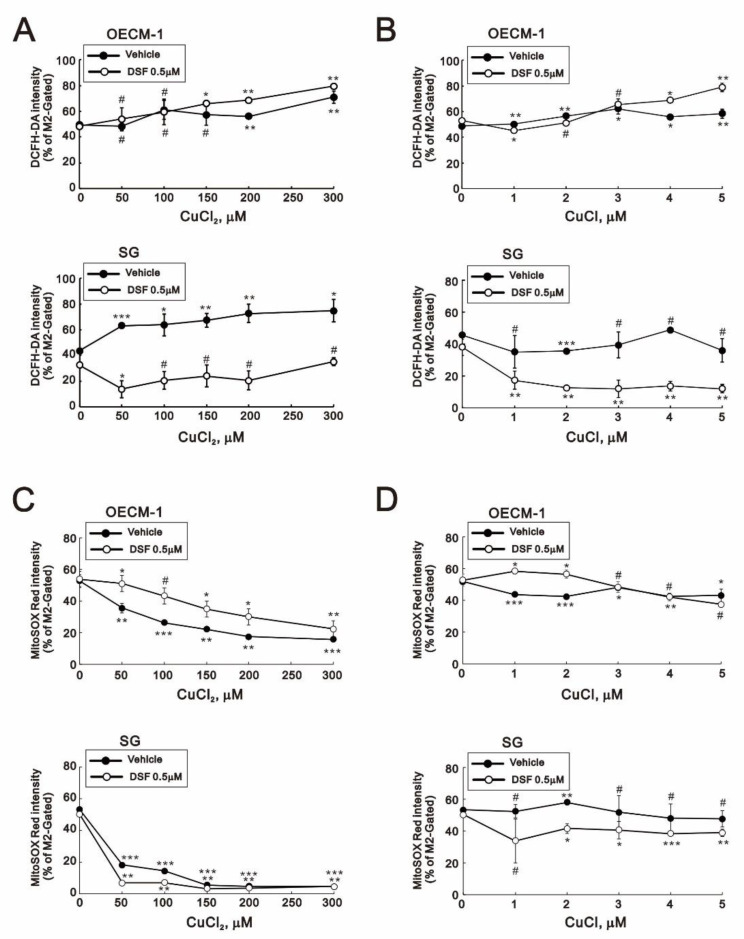
Effects of DSF, copper ions, and DSF-copper complexes on cytosolic and mitochondrial ROS in OECM-1 and SG cells. (**A**–**D**) OECM-1 (**A**,**C**) and SG (**B**,**D**) cells were treated for 2 h or 4 h with the indicated concentrations of Cu^+^ or Cu^2+^ in the absence or presence of 0.5 μM DSF, after which the live cells were stained with (**A**,**B**) 10 μM DCFH-DA for cytosolic ROS and (**C**,**D**) 5 μM MitoSOX red for mitochondrial ROS and assayed using a flow cytometer. The results are representative of three independent experiments. # *p* > 0.05, * *p* < 0.05, ** *p* < 0.01, and *** *p* < 0.001.

We next assessed cytosolic and mitochondrial ROS levels using DCFH-DA and MitoSOX, respectively (Figure 5). The combination of DSF with CuCl_2_ or CuCl dramatically reduced the cytosolic ROS (shown as % of M2 gated) in SG cells, whereas no change was observed in OECM-1 cells (Figure 5A,B). On the other hand, CuCl_2_ alone or in combination with DSF dramatically decreased mitochondrial ROS in both OECM-1 and SG cells (Figure 5C,D). Cancer cells upregulate their antioxidant capacity to maintain appropriate redox dynamics, despite high ROS levels. However, we found that levels of superoxide dismutase (SOD)1, SOD2, SOD3, catalase, and NRF2 were unchanged in the two cell types (data not shown).

### 2.3. Effects of Copper, DSF, and DSF-Cu^+^/Cu^2+^ Complexes on Mitochondrial Function in OECM-1 and SG Cells

**Figure 6:**
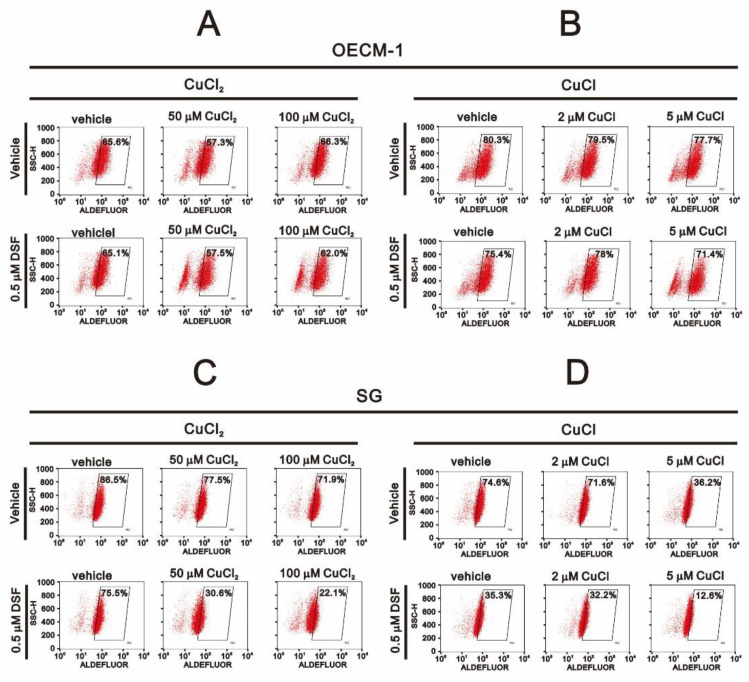
Effects of DSF, copper ions, and DSF-copper complexes on ALDH enzyme activity in OECM-1 and SG cells. (**A**,**B**) OECM-1 and (**C**,**D**) SG cells (2 × 10^5^ cells/well) were treated for 2 h or 4 h with the indicated concentrations of Cu^+^ or Cu^2+^ in the absence or presence of 0.5 μM DSF, after which the live cells were stained with ALDEFLUOR™ reagent and assayed using a flow cytometer. The results are representative of three independent experiments.

DSF is a well-known irreversible inhibitor of ALDH enzyme activity. We therefore applied Aldefluor aldehyde dehydrogenase activity analysis to verify irreversible ALDH inhibition by DSF in OECM-1 and SG cells (Figure 6). Our data showed that CuCl alone or the combination of DSF with CuCl or CuCl_2_ dramatically suppressed ALDH activity in SG cells (Figure 6C,D) but not OECM-1 cells (Figure 6A,B). Hence, DSF-induced ALDH inhibition was only confirmed and may play a protective role in SG cells.

**Figure 7:**
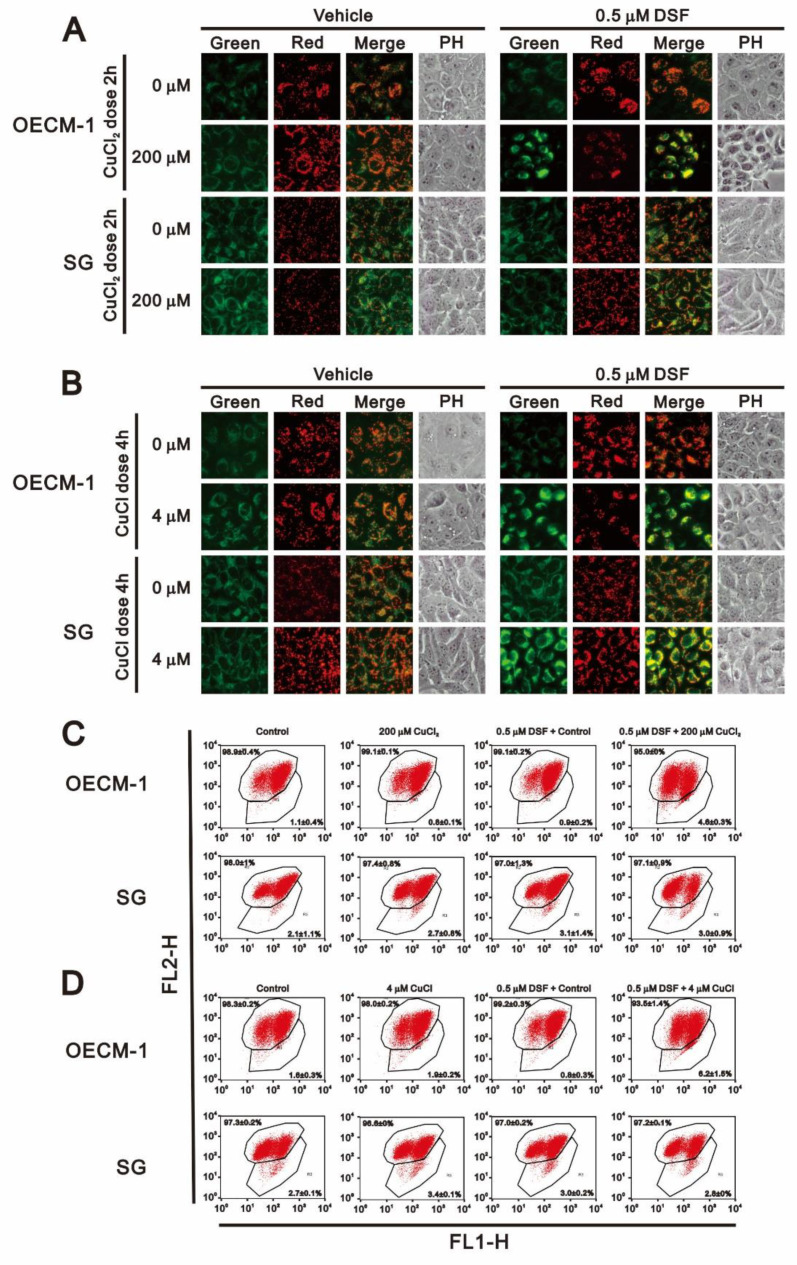
Effects of DSF, copper ions, and DSF-copper complexes on mitochondrial membrane potential in OECM-1 and SG cells. OECM-1 and SG cells were treated for 2 h or 4 h with the indicated concentrations of Cu^+^ or Cu^2+^ in the absence or presence of 0.5 μM DSF, after which the live cells were stained with 3–5 μM JC-1 dye. Images (**A**,**B**) were observed under a LeadView 2800AC-FL microscope equipped with a 40× objective, and effects on mitochondrial membrane potential (**C**,**D**) were assessed with a flow cytometer. The results are representative of three independent experiments.

**Figure 8:**
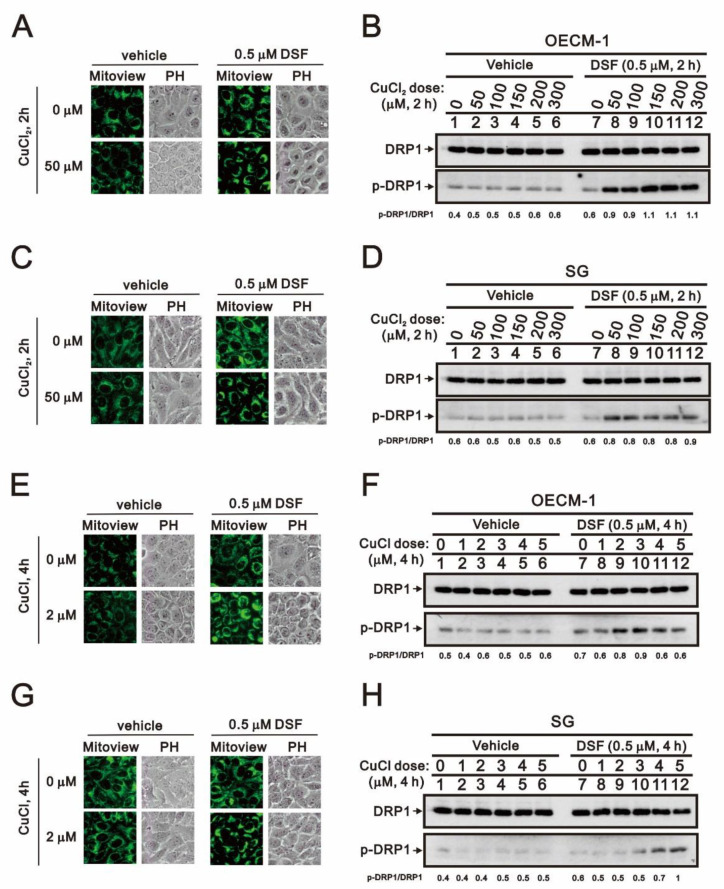
Effects of DSF, copper ions, and DSF-copper complexes on mitochondrial fission/fusion in OECM-1 and SG cells. OECM-1 and SG cells were treated for 2 h or 4 h with the indicated concentrations of Cu^+^ or Cu^2+^ in the absence or presence of 0.5 μM DSF, after which the live cells were stained with (**A**,**C**,**E**,**G**) 10 nM MitoView™. Cell lysates were subjected to Western blot analysis (**B**,**D**,**F**,**H**) using antibodies against DRP1 and p-DRP1. The results are representative of three independent experiments.

**Figure 9:**
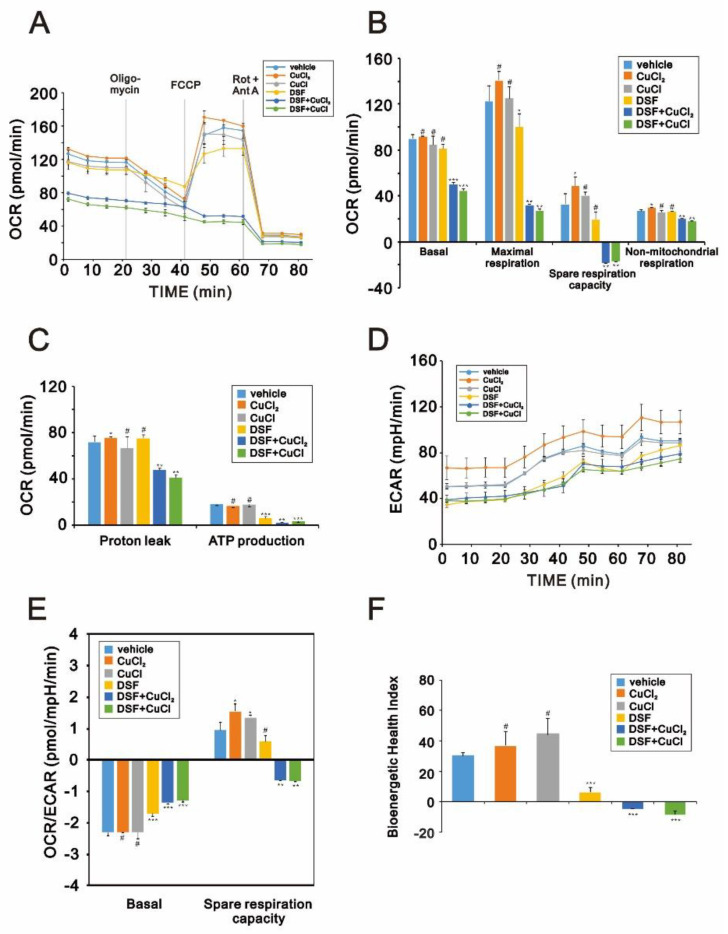
Effects of DSF, copper ions, and DSF-copper complexes on the oxygen consumption rate (OCR) and extracellular acidification rate (ECAR) in OECM-1 cells. (**A**–**D**) OECM-1 cells were treated for 2 h or 4 h with 0.5 μM DSF complexed with 5 μM Cu^+^ or Cu^2+^, after which cellular OCR (**A**–**C**), ECAR (**D**), OCR/ECAR (**E**), and the bioenergetic health index (**F**) were measured in XF24 bioenergetic assays. # *p* > 0.05, * *p* < 0.05, ** *p* < 0.01, and *** *p* < 0.001.

Having observed that DSF induces apoptosis and ROS generation, to further investigate the potential cytotoxicity of DSF in OECM-1 cells, we focused on its effects on mitochondrial function. Using the fluorescent voltage-sensitive dye JC-1, we first found that DSF reduced mitochondrial membrane potential (Figure 7) and that larger depolarizations were induced when DSF was applied in combination with CuCl or CuCl_2_. We then used fluorescent MitoView dye to observe the fission-fusion phase switch, which we verified based on the p-dynamin related protein 1 (p-DRP1)/DRP1 ratio detected through Western blotting (Figure 8). Our data suggested that in the presence of the combination of DSF with CuCl_2_, mitochondria showed more extensive fission in OECM-1 cells than SG cells. Using the Seahorse system, we also analyzed the effects of copper, DSF, and their combination on respiration and glycolysis pathways in OECM-1 cells (Figure 9). CuCl_2_ alone increased the OCR and ECAR rates, but the combination of DSF with CuCl_2_ or CuCl had the opposite effect (Figure 9A–D). DSF complexed with Cu^2+^ or Cu^+^ also suppressed proton leak, ATP production, the OCR/ECAR ratio (Figure 9E), and the bioenergetic health index (BHI) (Figure 9F). DSF alone decreased ATP production, the ECAR rate and the BHI.

### 2.4. Effects of Copper, DSF and DSF-Cu^+^/Cu^2+^ Complexes on the Expression of HIF-1α and c-Myc in OECM-1 and SG Cells

**Figure 10:**
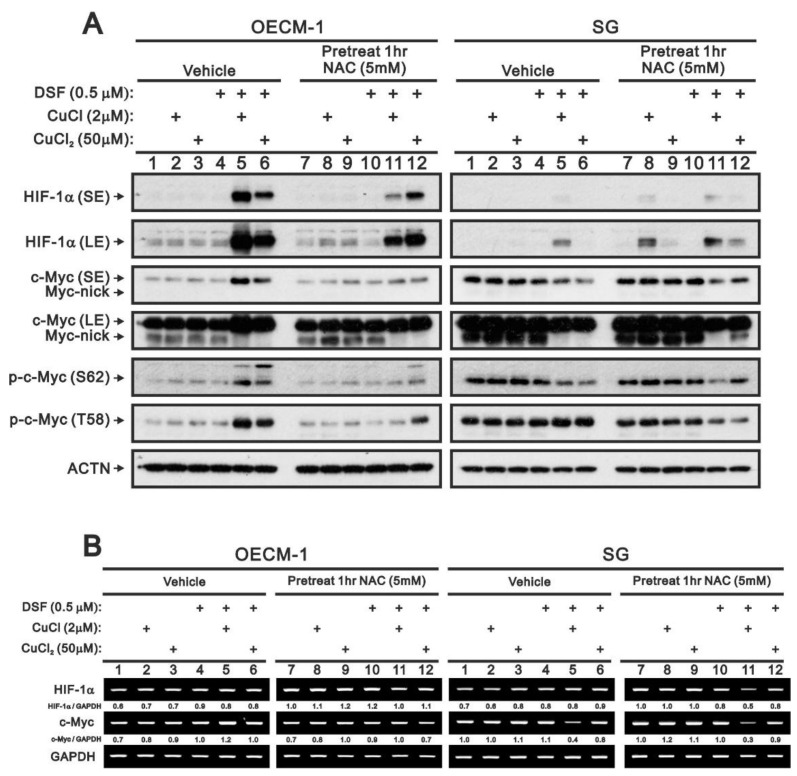
Effect of ROS on HIF-1α and c-Myc expression in OECM-1 and SG cells. OECM-1 and SG cells were treated for 2 h or 4 h with the indicated concentrations of Cu^+^ or Cu^2+^ complexed in the absence or presence of 0.5 μM DSF with or without pretreatment for 1 h with 5 mM NAC. (**A**) Western blot analysis for HIF-1α and c-Myc (total, nick, phosphorylated at T58 or S62) protein expression. (**B**) RT-PCR analysis for HIF-1α and c-Myc mRNA expression. ACTN was the protein loading control; GAPDH mRNA was the mRNA loading control. The results are representative of three independent experiments. Protein and PCR bands were quantified through pixel density scanning and evaluated using ImageJ, version 1.44, 31 January 2011 (http://imagej.nih.gov/ij/ (accessed on 15 March 2021)). The fold change (shown above the bands) was calculated by normalization to the internal control protein (ACTN) or gene (GAPDH).

We previously reported that ROS production may result in stabilization of HIF-1α [31]. To determine whether that occurs in OECM-1 or SG cells, we assessed the effects of the ROS scavenger NAC (Figure 10). Western blotting and RT-PCR analysis showed that the combination of DSF with CuCl_2_ or CuCl stabilized HIF-1α in OECM-1 cells to a much greater degree than in SG cells (Figure 10A). Pretreatment with NAC selectively suppressed the effects of DSF in combination CuCl, but not CuCl_2_, in OECM-1 cells, whereas the combination of DSF with CuCl_2_ or CuCl alone induced the stabilization of HIF-1α in SG cells. No effect on HIF-1α mRNA expression in OECM-1 or SG cells was detected (Figure 10B).

We and others previously reported that cellular stress enhances c-Myc degradation [32,33]. We therefore examined the status of c-Myc with respect to its stability, percentage truncated and phosphorylation at threonine 58 (T58) and serine 62 (S62). Western blotting showed that in OECM-1 cells the combination of DSF with CuCl_2_ or CuCl increased levels of full-length c-Myc protein as well as its phosphorylated forms and decreased the truncated form (Figure 10A). Pretreatment with NAC selectively reversed the effects of DSF with CuCl, but not CuCl_2_. Expression of HIF-1α and c-Myc mRNAs was unchanged by NAC in OECM-1 cells (Figure 10B). In SG cells, the combination of DSF with CuCl_2_ or CuCl decreased levels of both full-length and truncated c-Myc as well as the form phosphorylated at S62, but not at T58. Pretreatment with NAC failed to reverse the abovementioned effects. Instead, NAC enhanced the decreases in the T58 phosphorylated form of c-Myc and the HIF-1α and c-Myc mRNAs induced by DSF with CuCl. In addition, cycloheximide pulse-chase analysis showed that DSF in combination with CuCl_2_ or CuCl increased c-Myc stability in OECM-1 cells (data not shown).

**Figure 11:**
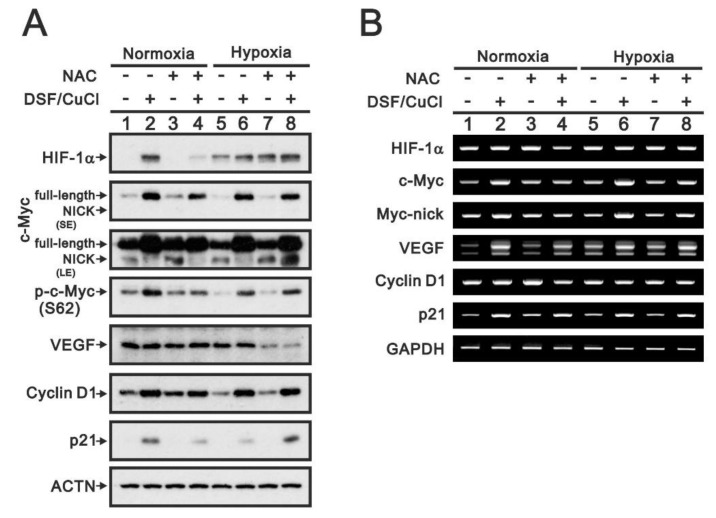
Functional roles of HIF-1α induced by DSF-CuCl under normoxia or hypoxia in OECM-1 cells. OECM-1 cells were treated for 4 h with 0.5 μM DSF/2 μM CuCl under normoxia or hypoxia with or without pretreatment for 1 h with 5 mM NAC. (**A**) Western blot analysis for HIF-1α and c-Myc (total, nick, or phosphorylated at S62), VEGF, cyclin D1, and p21 protein expression. (**B**) RT-PCR analysis for HIF-1α, c-Myc, VEGP, cyclin D1, and p21 mRNA expression. ACTN was the protein loading control; GAPDH mRNA was the mRNA loading control. LE: longer exposure; SE: shorter exposure.

To investigate the potential function of HIF-1α stabilized by the DSF-Cu^+^/Cu^2+^ complexes, we compared the effects of the complex with those of hypoxia (Figure 11). Consistent with hypoxia, the DSF/copper complex stabilized HIF-1α protein but had no effect on HIF-1α mRNA. However, the DSF-Cu^+^/Cu^2+^ complexes induced expression of c-Myc, VEGF, cyclin D1 and p21 proteins, and mRNAs in both normoxia and hypoxia. Pretreatment with NAC selectively suppressed the induction of HIF-1α, c-Myc, p-c-Myc (S62), cyclin D1, and p21 proteins, and mRNAs in normoxia. In hypoxia, NAC failed to suppress induction of HIF-1α, c-Myc, or cyclin D1 proteins and increased levels of truncated c-Myc and p21 proteins while decreasing VEGF protein (Figure 11A). No suppressive effect of NAC on HIF-1α, c-Myc, VEGF, cyclin D1, or p21 mRNAs was detected (Figure 11B).

**Figure 12:**
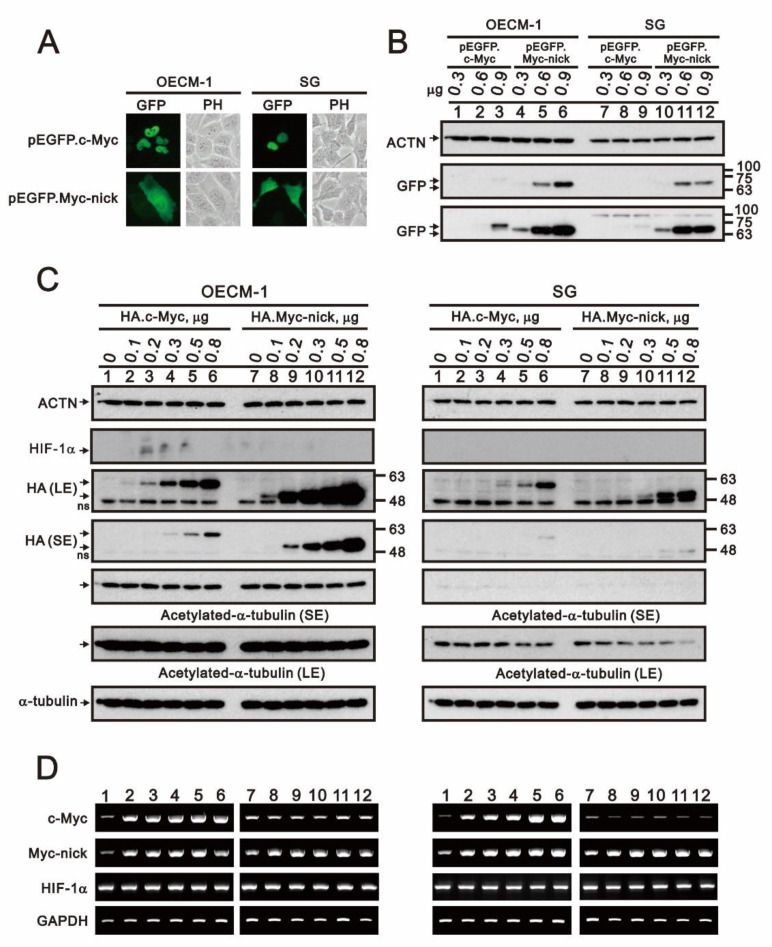
Subcellular localization and functional role of c-Myc (total and Nick) in OECM-1 and SG cells. OECM-1 and SG cells were transiently transfected with indicated amount of pEGFP.c-Myc (total or Nick) or pSG5.HA.c-Myc (total or Nick) expression plasmid. (**A**) Subcellular localization of total or nick EGFP.c-Myc protein observed under a LeadView 2800AC-FL microscope equipped with a 40× objective. (**B**,**C**) Western blot analysis of HIF-1α and c-Myc (total and nick) and total or acetylated α-tubulin. (**D**) RT-PCR analysis for HIF-1α and total or nick c-Myc mRNA. α-Tubulin was the protein loading control; GAPDH mRNA was the mRNA loading control. LE: longer exposure; SE: shorter exposure.

Because we detected expression of HIF-1α and c-Myc in OECM-1 cells, we transfected cells with full-length and nick truncated c-Myc pSG5.HA and pEGFP expression vectors to determine whether c-Myc is involved into the regulation of HIF-1α expression (Figure 12). We observed that while full-length c-Myc was localized in the nucleus, nick truncated c-Myc was in the cytosol (Figure 12A). The expression level of nick truncated c-Myc protein was higher than full-length c-Myc protein (Figure 12B,C). Expression of both c-Myc proteins was higher in OECM-1 than SG cells. The levels of exogenously transfected DNA were confirmed using RT-PCR (Figure 12D). Previous studies demonstrated that nick truncated c-Myc may induce acetylation of cytosolic α-tubulin [34,35]. However, we detected no change the levels of acetylated α-tubulin in OECM-1 cells and a decrease in SG cells. Neither c-Myc protein had an apparent effect on the expression of HIF-1α mRNA or protein, which suggests c-Myc may not be the major transcription factor governing HIF-1α expression in OECM-1 and SG cells.

## 3. Discussion

By suppressing various cancer-associated pathways mediated through ROS, ALDH and others, the anti-alcoholism drug DSF may exert potentially beneficial effects in OSCC. In addition to its inhibitory effects on ALDH, DSF also acts as a metal chelator that primarily complexes with Cu^2+^. In the present study, we complexed DSF with Cu^+^ or Cu^2+^ to examine their effects on OECM-1 oral cancer cells. Our findings show that the primary antitumor activity of the DSF-Cu^2+^ complex reflected multiple effects on the cell cycle, cellular proliferation, cellular senescence, ROS, mitochondrial function (membrane potential, fission-fusion, OCR, ECAR, ATP production, and BHI, and ROS-induced HIF-1α and c-Myc expression. Notably, the DSF-Cu^2+^ complex failed to inhibit ALDH enzyme activity in OECM-1 cells, though it did inhibit the enzyme in SG normal gingival epithelial cells. These findings suggest that the DSF-Cu^2+^ complex is more effective than the DSF-Cu^+^ complex when applied as a repurposed agent for treatment of OSCC patients.

Our results indicate that CuCl, DSF and the DSF-Cu^+^/Cu^2+^ complexes all inhibited ALDH activity in SG cells but not OECM-1 cells, though the reason for the different cellular responses is unclear. DSF-Cu^+^/Cu^2+^ complexes exhibited less cytotoxicity toward SG cells than OECM-1 cells, suggesting that ALDH activity is disadvantageous for normal cell survival. Cancer stem cell biomarkers for OSCC include ALDH, especially the ALDH1 isoform. ALDH1-positive cells exhibit radio-resistance and co-express Snail, which is evidence of epithelial-mesenchymal transition with a resultant increase in tumorigenicity. The ALDEFLUOR^®^ assay system was developed to detect the activity of the ALDH1 isoform. Here, CuCl_2_, CuCl, DSF and DSF-Cu^+^/Cu^2+^ complexes all failed to inhibit ALDH activity in OECM-1 cells. The reason for the differential effects between normal and tumor cells remains unclear. One possibility is that the concentrations used were not sufficient to inhibit the enzyme in OECM-1 cells as they did in SG cells. However, our findings suggest that the contribution of ALDH to cell survival differs between normal and tumor cells and that the mechanism by which the DSF-Cu complex exerts its effects is something other than inhibition of ALDH activity.

DSF in complex with copper has been shown to reverse drug resistance in cancer through its targeting of ALDH, MAPK and NF-κB, among others [16]. Production of ROS in response to DSF-copper treatment precedes the induction of apoptosis in melanoma [36]. The DSF-copper complex also potently inhibits proteasomal activity in MDA-MB-231 breast cancer cells, before induction of apoptotic cell death [37]. In that study, it was also revealed that the DSF-copper complex mediates phosphorylation of AMPKα1 and ERK in both OECM-1 and SG cells, which suggests an AMPKα1- or ERK-dependent pathway is not primarily responsible for the cytotoxicity of the DSF-copper complex in OSCC.

We used a relatively low dosage of DSF (0.5 μM vs. normal 20 μM) to highlight the combination of DSF with Cu^+^ or Cu^2+^. At that dosage, DSF induced apoptosis, mitochondrial dysfunction, suppression of cellular proliferation and stabilization of HIF-1α in OECM-1 cells only when complexed with copper. Differences in ROS generation between OECM-1 and SG cells could be the primary driver for the differences in the responses of these cells to DSF. If so, pretreatment with NAC should rescue OECM-1 cells from DSF-Cu^+^/Cu^2+^ complex-induced cell death. In addition, because DSF is a highly specific Cu^2+^ chelator, it could perhaps be applied to modulate copper homeostatic pathways or copper-induced oxidative stress in neurodegenerative conditions, like Alzheimer’s disease, or copper-overload disorders, like Wilson’s disease [38]. Cu^2+^-induced ROS may inhibit pyruvate dehydrogenase and alpha-ketoglutarate dehydrogenase resulting in neuronal and hepatocellular death [39].

Ferrous iron and 2-oxoglutarate-dependent oxygenases are thought to be involved in the post-translational modification of some proteins. This includes collagen C-4 prolyl hydroxylation, HIF-1α prolyl hydroxylation and histone demethylation [40]. In those cases, copper ions could potentially displace ferrous iron from their cognate ligands in prolyl hydroxylases for HIF-1α protein degradation. Here, the DSF-Cu^+^/Cu^2+^ complexes significantly stabilized HIF-1α protein in OECM-1 cells, but not SG cells. Further, the DSF-Cu^+^/Cu^2+^ complexes significantly induced c-Myc transcription, translation, phosphorylation at T58 and S62, and stability. However, they suppressed the nick form of c-Myc, suggesting post-translation modification is involved in the regulation of c-Myc. In our overexpression experiment, c-Myc had no ability to induce or stabilize HIF-1α. However, the details of the relationship between HIF-1α and c-Myc in OSCC remain to be investigated.

The DSF-Cu^2+^ complex had better antitumor activity than the DSF-Cu^+^ complex in OECM-1 cells. This may reflect the difference in the redox statuses of CuCl_2_ and CuCl in OECM-1 cells. In addition, DSF can act as a coordinating agent to copper ions, especially to Cu^+^, because Cu^+^ is a soft Lewis acid and DSF is a soft Lewis base [41]. Future experiments will require a selective chelator or indicator to differentiate between Cu^2+^ and Cu^+^ to explore copper ion regulation in OSCC cells and the relative contributions of metal competition, ROS generation, ALDH inhibition, and mitochondrial dysfunction to the anticancer activity of the DSF-Cu^+^/Cu^2+^ complexes.

## 4. Materials and Methods

### 4.1. Cell Culture and Chemicals

For this study, oral epidermoid carcinoma meng-1 (OECM-1) cells, originally derived from a primary culture of cells from an oral squamous carcinoma patient by Professor C.L. Meng, were compared with human Smulow-Glickman (SG) normal gingival epithelial cells [30]. OECM-1 and SG cells were cultured in Roswell Park Memorial Institute (RPMI) 1640 (Corning, USA) medium supplemented with 10% fetal bovine serum (FBS) and 1% penicillin-streptomycin (Thermo Fisher Scientific, CA, USA). *N*-acetyl cysteine (NAC), copper (II) chloride (CuCl_2_), 2′,7-dichlorofluorescein diacetate (DCFH-DA), disulfiram (DSF), propidium iodide (PI), and thiazolyl blue tetrazlium bromide (MTT) were obtained from Sigma Aldrich (St. Louis, MO, USA). Copper (I) chloride (CuCl) was from Alfa Aesar (Tewksbury, MA, USA).

### 4.2. Cell Survival Analysis

OECM-1 and SG cells were plated in 24-well plates and cultured in the presence of the indicated drugs. The cells were then incubated with MTT solution for 1 h at 37 °C, after which dimethyl sulfoxide (200 μL) was added, and the absorbances at 570 nm and 650 nm were measured using an ELISA plate reader (Multiskan EX, Thermo, Waltham, MA, USA). The control group, containing cells cultured in medium only, was defined as 100% cell survival.

### 4.3. Fluorescence-Activated Cell Sorting (FACS), Cell Cycle Profiles, Apoptosis, Cytosolic/Mitochondrial ROS, and Senescence Analyses

Cell cycle profiles were evaluated based on cellular DNA content using FACS. Cells (5 × 10^4^ cells/well) were fixed in 70% ice-cold ethanol and stored at −30 °C overnight, after which they were washed twice with ice-cold PBS supplemented with 1% FBS and stained with propidium iodide (PI) solution (5 μg/mL PI in PBS, 0.5% Triton x-100, and 0.5 μg/mL RNase A) for 30 min at 37 °C in the dark.

Early- and late-stage apoptotic cells were evaluated using a fluorescein phycoerythrin (PE)-Annexin V Apoptosis Detection Kit (BD Biosciences, Franklin Lakes, NJ, USA) according to the manufacturer’s protocol. Cells (5 × 10^4^ cells/well) were stained with PE-Annexin V as well as 7-amino-actinomycin (7-AAD), which enabled identification early apoptotic cells. Viable cells were PE Annexin V and 7-AAD negative; early apoptotic cells were PE Annexin V positive and 7-AAD negative; and late apoptotic and dead cells were both PE Annexin V and 7-AAD positive.

The fluorescent markers DCFH-DA and MitoSOX™ Red (Invitrogen, Carlsbad, CA, USA) were used to determine the intracellular and mitochondrial ROS levels, respectively. Cells (5 × 10^4^ cells/well) were incubated for the indicated times with different combinations of DSF-Cu^+^/Cu^2+^ complexes. Living cells were then stained with DCFH-DA (20 μM) and MitoSOX™ (5 μM) Red for 40 min at 37 °C and harvested. After washing the cells once with PBS, they were evaluated using a FACSCalibur flow cytometer and Cell Quest Pro software (BD Biosciences, Franklin Lakes, NJ, USA).

For flow cytometric senescence assays, senescence-associated β-Gal activity was measured using the fluorescent substrate 5-dodecanoylaminofluorescein di-β-d-galactopyranoside (C12FDG), (Invitrogen, Carlsbad, CA, USA) according to the manufacturer’s instructions. Briefly, cells (5 × 10^4^ cells/well) were seeded into 6-well culture plates and treated for the indicated times with different combinations of DSF-Cu^+^/Cu^2+^ complexes. After the incubation, the cells were harvested, washed twice with PBS, and stained with 33 μM C12FDG for 15–20 min at room temperature. Fluorescence intensity was then evaluated using a FACSCalibur flow cytometer and Cell Quest Pro software (BD Biosciences, Franklin Lakes, NJ, USA).

### 4.4. Western Blotting

Drug-treated OECM-1 and SG cells were lysed in RIPA buffer (100 mM Tris-HCl (pH 8.0), 150 mM NaCl, 0.1% SDS, and 1% Triton 100) at 4 °C. Proteins in the resultant lysates were separated by SDS-PAGE and analyzed by immunoblotting with antibodies against α-actinin (ACTN), α-tubulin, acetylated-α-tubulin, DRP1, GFP, VEGF (Santa Cruz Biotechnology, Santa Cruz, CA, USA), c-Myc/N-Myc, phospho-c-Myc (Ser62), phospho-c-Myc (Thr58), p-DRP1, HIF-1α (Cell Signaling, Danvers, MA, USA), cyclin D1 and p21 (Abcam, Cambridge, UK).

### 4.5. Evaluation of Mitochondrial Morphology

OECM-1 and SG cells were seeded into 12-well plates at a density of 10^5^ cells/well. After treating the cells for the indicated times with selected combinations of DSF-Cu^+^/Cu^2+^ complexes, the cells were washed, incubated with 10 nM MitoView™ Green (Biotium, Fremont, CA, USA) at 37 °C for 15 min, and washed again three times with PBS. Mitochondrial morphology in each group was observed using a LeadView 2800AC-FL microscope (Leader Scientific Co. Ltd., Taiwan, ROC) equipped with a 40× objective and analyzed using Image-Pro^®^Plus (Media-cybernetics, Rockville, MD, USA) [42]. Green fluorescence revealed the mitochondria stained by MitoView™ Green.

### 4.6. Reverse Transcription-Polymerase Chain Reaction (RT-PCR)

Total RNA was isolated from OECM-1 and SG (5 × 10^4^ cells/well) cells using TRIzol reagent (Invitrogen). Reverse transcription for first strand cDNA synthesis was conducted using MMLV reverse transcriptase (Epicentre Biotechnologies, Madison, WI, USA) with 1 μg of total RNA for 60 min at 37 °C. PCR reactions were run in a GeneAmp PCR system 9700 (Biometra, Göttingen, Germany). The PCR primers are listed in Table 2.

### 4.7. Transient Transfection and Determination of Subcellular Localization and Expression Levels

OECM-1 and SG cells (5 × 10^4^ cells/well) were plated in 6-well plates, and 1.0 μg of reporter was transfected using jetPEI (Polyplus Transfection Inc., New York, NY, USA). Transient transfection of pEGFP fusion proteins was observed after 16 h using a LeadView 2800AC-FL microscope (Leader Scientific Co. Ltd., Taiwan, ROC) equipped with a 40× objective.

### 4.8. Detection of the Oxygen Consumption Rate (OCR) and Extracellular Acidification Rate (ECAR)

The cellular OCR and ECAR were measured using a Seahorse XF24 bioenergetic assay according to the manufacturer’s instructions (Agilent, Santa Clara, CA, USA). Procedural details were described previously [43,44]. Briefly, OECM-1 cells (5 × 10^3^ cells/well) were plated on an XF24 microplate and cultured for 3 days. Thereafter, XF24 bioenergetic assays were started by replacing the exhausted medium with sodium bicarbonate-free Dulbecco’s modified Eagle medium (DMEM; pH 7.4) supplemented with 2% FBS and 2% horse serum. The OCR and ECAR were measured at steady state, after which oligomycin (1 μM), carbonyl cyanide 4-[trifluoromethoxy] phenylhydrazone (FCCP; 0.5 μM) and rotenone/antimycin (0.5 μM) were added sequentially to the wells to obtain values for the maximal and non-mitochondrial respiration rates.

### 4.9. Statistical Analysis

Values are expressed as the mean ± SD of at least three independent experiments. All comparisons between groups were made using Student’s *t*-tests. Statistical significance was set at *p* < 0.05.

## Figures and Tables

**Table 1:** The effects of DSF-copper complexes on the cell cycle profile of OECM-1 and SG cells.

**OECM-1 Cells**						
	**Vehicle**			**0.5 μM DSF**		
	Vehicle	CuCl	CuCl_2_	Vehicle	CuCl	CuCl_2_
SubG1 (%)	0.9 ± 0.01	0.8 ± 0.01 ^#^	1.6 ± 0.2 *	0.8 ± 0.04 ^#^	22.6 ± 1.3 ***	30.8 ± 0.9 ***
G1 (%)	50.1 ± 0.3	52.2 ± 0.8 *	45.1 ± 1.2 **	54.7 ± 0.1 ***	47.1 ± 0.9 **	48.4 ± 1.2 *
S (%)	15.6 ± 0.7	12.9 ± 0.2 **	21.4 ± 1.8 **	14.6 ± 0.4 ^#^	2.7 ± 0.1 ***	2.1 ± 0.1 ***
G2/M (%)	31.0 ± 0.5	31.8 ± 1.1 ^#^	29.4 ± 3 ^#^	27.2 ± 0.4 ***	21.6 ± 0.3 ***	13.6 ± 0.04 ***
**SG Cells**						
	**Vehicle**			**0.5 μM DSF**		
SubG1 (%)	0.3 ± 0.1	0.3 ± 0.1 ^#^	0.5 ± 0.1 *	0.3 ± 0.2 ^#^	0.5 ± 0.1 *	1.0 ± 0.4 *
G1 (%)	50.5 ± 0.5	49.6 ± 1.4 ^#^	47.1 ± 3.3 ^#^	50.5 ± 1.9 ^#^	54.5 ± 1.7 *	59.0 ± 0.8 ***
S (%)	39.7 ± 1.2	40.8 ± 0.8 ^#^	43.3 ± 3.2 ^#^	40.1 ± 2.4 ^#^	22.1 ± 2.7 ***	5.8 ± 1.1 **
G2/M (%)	8.2 ± 0.2	8.3 ± 0.2 ^#^	8.4 ± 0.2 ^#^	8.2 ± 0.5 ^#^	17.1 ± 1.7 **	24.9 ± 6.0 *

# *p* > 0.05, * *p* < 0.05, ** *p* < 0.01, and *** *p* < 0.001.

**Table 2:** PCR primers used in this study.

Gene Name	Primer Sequence (5′ → 3′)
*cyclin D1*	Forward: 5′-ATGGAACACCAGCTCC-3′ Reverse: 5′-TCAGATGTCCACGTCCCGC-3′
*c-Myc* (+*1/−1320*)	Forward: 5′-AAGAATTCATGCCCCTCAACGTTAGCTTC-3′ Reverse: 5′-AACTCGAGTTACGCACAAGAGTTCCGTAG-3′
*GAPDH*	Forward: 5′-CTTCATTGACCTCAACTAC-3′ Reverse: 5′-GCCATCCACAGTCTTCTG-3′
*p21*	Forward: 5′-CTGAGCCGCGACTGTGATGCG-3′ Reverse: 5′-GGTCTGCCGCCGTTTTCGACC-3′
*Myc-nick* (+*1/−894*)	Forward: 5′-AAGAATTCATGCCCCTCAACGTTAGCTTC-3′ Reverse: 5′-AACTCGAGTTACTTGAGGACCAGTGGGC-3′
*HIF-1α*	Forward: 5′-GAACCTGATGCTTTAACT-3′ Reverse: 5′-CAACTGATCGAAGGAACG-3′
*VEGF*	Forward: 5′-GGACATCTTCCAGGAGTACC-3′ Reverse: 5′-GTTCCCGAAACCCTGAGGG-3′

## Abbreviations

7-AAD	7-Amino-Actinomycin
ACTN	α-actinin
ALDH	Aldehyde dehydrogenase
BHI	Bioenergetic health index
BrdU	5-bromo-2-deoxyuridine
C12FDG	5-dodecanoylaminofluorescein di-β-d-galactopyranoside
CHX	cycloheximide
DCFH-DA	2′,7-dichlorofluorescein diacetate
DMEM	Dulbecco’s modified Eagle’s medium
DRP1	Dynamin related protein 1-
DSF	Disulfiram
FACS	Fluorescence-activated cell sorting
FBS	Fetal bovine serum
FCCP	Carbonyl cyanide 4-(trifluoromethoxy) phenylhydrazone
FITC	fluorescein isothiocyanate
H_2_O_2_	Hydrogen peroxide
HIF-1α	Hypoxia-inducible factor 1 alpha
JC-1	5,5′,6,6′-tetrachloro-1,1′,3,3′-tetraethylbenzimi-dazolyl carbo-cyanine iodide
LC3B	Microtubule-associated protein 1 light chain 3B
MTT	Thiazolyl blue tetrazlium bromide
NAC	*N*-acetyl cysteine
OCR	Oxygen consumption rate
OECM-1	Oral epidermoid carcinoma meng-1
OSCC	Oral squamous cell carcinoma
PBS	Phosphate buffered saline
PE	phycoerythrin
PI	Propidium iodide
RPMI	Roswell Park Memorial Institute
ROS	Reactive oxygen species
RT-PCR	Reverse transcription-polymerase chain reaction
SG	Smulow-Glickman
SOD1	Superoxide dismutase 1
